# The impact of the Covid-19 pandemic on primary health care utilization: an experience from Iran

**DOI:** 10.1186/s12913-022-07753-5

**Published:** 2022-03-27

**Authors:** Ramin Rezapour, Abbas Ali Dorosti, Mostafa Farahbakhsh, Saber Azami-aghdash, Ilnaz Iranzad

**Affiliations:** 1 Student Research Committee, Tabriz University of Medical Sciences, Tabriz, Iran; 2 Department of Anaesthesiology, Tabriz University of Medical Sciences, Tabriz, Iran; 3 Clinical Psychiatry Research Center, Tabriz University of Medical Sciences, Tabriz, Iran; 4 Tabriz Health Services Management Research Center, Tabriz University of Medical Sciences, Tabriz, Iran; 5 Health Vice-Chancellor, Tabriz University of Medical Sciences, Tabriz, Iran

**Keywords:** Primary health care, Service utilization, Service delivery status, Covid-19, Assessment, Pandemic, Iran

## Abstract

**Background:**

The Covid-19 pandemic affected the performance of Primary Health Care (PHC) worldwide. This study was performed to investigate the impact of the Covid-19 pandemic on the utilization of PHC in Iran.

**Method:**

A before and after study conducted between 2019 and 2021. 56 medical science universities across the country were studied. The data extracted from Electronic Health Record (EHR) is entitled “SIB”. Three major indicators included a weighted average of essential services provided by (physician, dentist, mental health expert, midwife, nutritionist), percentage of actual delivered service, and percentage of customer satisfaction was selected as a criterion for assessing the PHC. Descriptive statistics and analytical statistics (Wilcoxon test) using SPSS 16 software were used for the data analyzing and reporting.

**Results:**

There was a significant difference among the examined dimensions before and after Covid-19 separation in all studied indicators except the level of percentage of customer satisfaction (*P* < 0.05). So that the percentage of actually delivered services decreased about 1% and the weighted average of essential services provided by a physician, dentist, midwife, mental health experts, and nutritionist decreased 627.95, 718.81, 460.85, 2914.66, 2410.65 numbers, respectively.

**Conclusion:**

Covid-19 Pandemic has affected the performance of Iranian PHC at the beginning and overall, has a negative consequence on utilization of services. Preparedness to respond to pandemics and develop programs and interventions is necessary to cover the weaknesses of the PHC.

**Supplementary Information:**

The online version contains supplementary material available at 10.1186/s12913-022-07753-5.

## Background

The Covid-19 was announced by the World Health Organization (WHO) on March 11, 2020 as an epidemic disease [1]. WHO considers the Covid-19 to be a cause for concern and danger for countries with weak and vulnerable health system [1]. One of the strategic goals in controlling the Covid-19 is to reduce its economic consequences for all countries and prevent the dissemination of misinformation, globally [2, 3].

Today decisions on health policy and resource allocation in response to the Covid-19 outbreak will shape our world for next year’s [4]. Since countries take emergency measures for meeting the immediate needs of the community and healthcare workers, identifying and addressing the main weaknesses in the health systems is also vital [4]. Primary Health Care (PHC) that can meet more than 80% of people’s health needs at any age and any stage of life can play a very important and vital role in these area [4].

Not only could strengthening PHC reduce the effects of the Covid-19 on millions of people’s health and well-being but also limits susceptibility to the next epidemic disease [5]. Strong PHC can be the first line of defense to maintain the safety and health of people [5]. PHC can help diagnose, track, and prevent local outbreaks by providing essential health services to communities [6]. PHC, as the first and most regular point of individuals’ contact with the health system, to recognize, track and report important cases is vital to help to reduce the rate of outbreaks within countries [6]. After the outbreak of SARS in the 2003 year, Taiwan invested enormous money so as to enhance the PHC and increase the motivation of health providers as the first responder to the disease and provide care to the communities [6]. During the Ebola outbreak in the 2014 year, Liberia trained and mobilized a network of community health workers to track and manage the disease in remote and rural areas; An important skill that now helps to prevent the spread of the Covid-19 [6, 7].

PHC plays a very important role, especially in Low and Middle-Income Countries (LMICs) that people may have limited access to hospitals and specialized care [5]. A recent study has shown that nearly 30% of people across Sub-Saharan Africa do not have access to emergency hospital care [8]. Meanwhile, countries are adopting various programs, interventions, and responses to deal with the Covid-19 pandemic [9]. The world cannot ignore the widespread health needs of the people, including vaccinations, healthy pregnancy, maternal and child care, HIV/AIDS services, tuberculosis and malaria, mental health, and chronic disease care [10]. Neglecting these needs will create a wider crisis and it will make millions of people vulnerable to preventable and treatable diseases [10].

When health systems are under too much pressure during an outbreak, deaths due to lack of providing or failing to provide routine care can increase dramatically [10, 11]. Studies estimate that impairments and poor performance of PHC during the Ebola outbreak in 2014–2016 in West Africa led to 10,000 extra preventable deaths due to malaria, HIV/AIDS, and tuberculosis [8]. Due to the Covid-19, polio, measles, cholera, yellow fever and meningitis vaccinations are currently has been postponed [8]. UNICEF has estimated that 117 million children in 37 countries are at risk for measles for saving their lives. There is a delay in 10% of orders for HIV/AIDS, tuberculosis, and malaria drugs by more than 30 days [8]. PHC staff are unable to deliver goods and anti-malaria services due to locks and applying social distance. 40 million children in Pakistan missed polio vaccination in April 2020 [8].

By committing to prioritizing PHC, countries can shape a future in which everyone has access to the care they need in all circumstances. Addressing the challenges of providing basic and essential services in response to the epidemic disease and its recovery, it needs strong and resilient health systems, especially at the PHC level. Generating and using better data helps managers to identify weak points in the PHC before assessments and emergency checks after Covid-19 pandemics and strengthen the provision of routine care.

The performance of the Iranian health system, like many countries in the world, was affected by the Covid-19 pandemic [12, 13]. Previous studies have reported challenges such as sustained workload among healthcare workers, inefficient management of equipment, and mental health problems as the main challenges of the Iranian health system [13, 14]. Reducing the rate of essential primary health services is also one of the most important challenges for health systems in the world [15, 16]. Hence, the study investigated the impact of the Covid-19 pandemic on the service utilization status in the Iranian PHC system.

## Method

This is a before and after study that was conducted between 2019 (March 2019 – Jan 2020) as before and 2021 (Feb 2020 – Feb 2021) as after. Data from the Electronic Health Record (EHR) entitled “SIB” was extracted and analyzed for the whole country. SIB system is a comprehensive information system in the field of PHC that provides all the information about the quantity and quality of services registered separately by service providers.

### Primary health care (PHC) in Iran

**Fig. 1:**
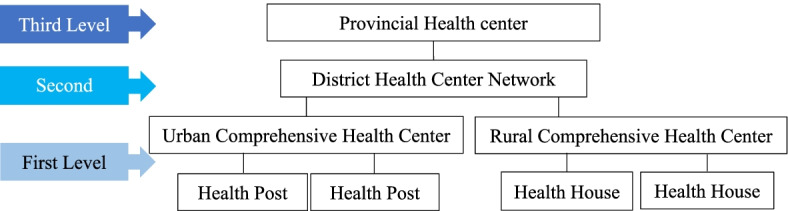
Iranian Primary Health Care System Structure

PHC is organized at three levels (Fig. 1). The first level consists of facilities where the first and widest contact of the community with the health care delivery system takes place in it. these facilities include health houses and health post, urban and rural comprehensive health service centers. Health workers working in health houses mainly include rural health workers (Behvarz) and in urban and rural comprehensive health service centers, usually including General Practitioners (GPs), sometimes dentists, nutritionists, mental health experts, and other health care providers [17, 18]. the second level of service delivery includes district health centers. A set of first and second-level facilities in the geographical area of ​​the city forms the district health canter network [19, 20]. The third level of service delivery is complementary to the second level. The provincial health center is located at this level. The movement of patients between the mentioned levels is done in the form of a referral system [21].

#### Selected indicators

To assess the status of service delivery in PHC three key indicators including the Actual Percentage of Service Delivery (APSD), Percentage of Customer Satisfaction (PCS), and weighted average of services by PHC providers (physician, dentist, midwife, nutritionist, and mental health expert) were used. Given that the quantity and quality of essential services provided are reflected in the calculation of these indicators, therefore examining and analyzing these indicators can be a good representative to evaluate the status of services provided in PHC.

#### Calculation of indicators

Actual Percentage of Service Delivery (APSD): This indicator shows the number of people who have gone to PHC facilities and have received the service. For each customer in PHC facilities a text message entitled “Has the service been received?” is sent. The customer’s response to the Short Message System (SMS) (approval or disapproval) is considered as the basis for calculating the indicator. Thus, the indicator is calculated using the following formula.

$$\mathrm{APSD}=\frac{Number\ of\ people\ who\ have\ confirmed\ receiving\ the\ service}{All\ people\ who\ have\ referred\ to\ PHC\ facilities}\times 100$$

Percentage of Customer Satisfaction (PCS): For each customer, a text message entitled “ Are you satisfied with the services provided? “The answer to this question is twofold (yes/no). The indicator is calculated through the following formula.

$$PCS=\frac{Number\ of\ people\ were\ satisfied\ with\ the\ services\ received}{All\ people\ who\ have\ received\ the\ service}\times 100$$

A weighted average of services: this indicator shows the number of essential services provided by each of the PHC providers. Each of the providers is required to provide a set of services based on their specialty (attachment1). the weighted average instead of a simple average was used to calculate this indicator. The indicator was calculated using the following formula.

$${A}_W=\frac{\sum_{i=1}^n\left(\ {s}_n\times {w}_n\right)}{N}$$

A_W_ = Weighted Average of services.

S_n_ = Number of Services.

W_n_ = Weights of Service.

N = Sum of Weights.

n = required set of services.

### Data analysis

To analyze the indicators data, first, the indicators were estimated monthly for 2019–2021. to describe the Data, the “average” index was used in the form of linear graphs. to examine the difference between the mean of the indicators in two time periods before and after the Covid-19, Analytical statistics including the Wilcoxon test was used. The significance level of 0.05 was considered as the confidence level. SPSS 16 software was used for data analysis.

## Results

The indicators were extracted and analyzed from 56 universities of medical sciences from 2019 to 2021. The results show that the crisis of Covid-19 has been impressive on the status of the PHC. There was a significant difference in the period of before and after Covid-19 in all studied indicators except the level of PCS. So that the number of indicators after the Covid-19 outbreak was significantly reduced (Table 1).

**Table 1:** Compare means of services number before and after COVID-19 in the Iranian PHC

Indicators	before (January 2019(	after (March 2020)	Mean Difference	*P* value
The actual percentage of service delivery	97.49	96.94	−0.55	0.003
physician services (Number)	3588.5	1177.85	−2410.65	*P* < 0.001
Dentist services (Number)	3248.94	334.28	−2914.66	*P* < 0.001
Midwifery services (Number)	1534.03	1073.18	−460.85	*P* < 0.001
mental health expert services (Number)	1579.55	860.74	−718.81	*P* < 0.001
Nutritionist services (Number)	802.57	174.62	−627.95	*P* < 0.001

### Time trend of actual service delivery percentage

**Fig. 2:**
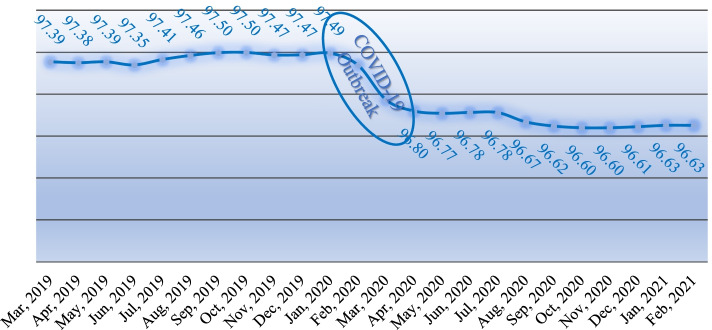
Actual Percentage of Service Delivery before and after COVID-19 in the Iranian PHC - Trend (2019–2021)

The results of time trend analysis show that the percentage of actual service delivery has been decreased after the occurrence of the Covid-19 pandemic (Fig. 2). The trend of this indicator has been relatively increased from the beginning of 2019 until the occurrence of the Covid-19 pandemic and no significant change was observed in the indicator trend (*P* > 0.05). As it is observed in Fig. 2, after the separation of the Covid-19 pandemic, the indicator trend has decreased and the percentage of actual service delivery has decreased about 1% (*P* = 0.003).

### Time trend of percentage of customer satisfaction (PCS)

**Fig. 3:**
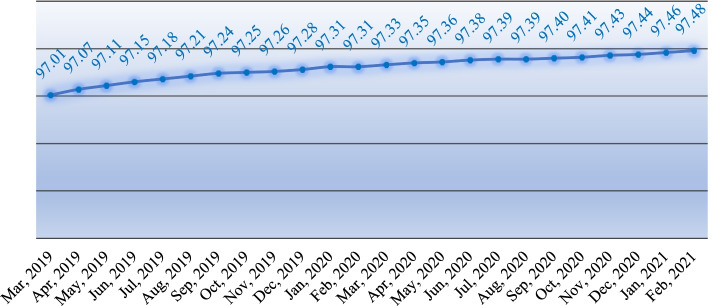
Customer Satisfaction Percentage before and after COVID-19 in the Iranian PHC - Trend (2019–2021)

The results of the analysis of PCS in the period of 2019–2021 show that the occurrence of the Covid-19 pandemic has no significant effect on the level of customer satisfaction (*P* > 0.05). Time trend analysis shows that the percentage of customer satisfaction has increased with a gentle slope from the beginning of 2019 to 2021, and the occurrence of the Covid-19 pandemic has not affected this process (Fig. 3).

### The weighted average number of services based on the provider

Based on the results of analysis of PHC provider performance in the period of 2019–2020, it was determined that the number of essential services provided after the occurrence of the Covid-19 pandemic (Jan 2020) has significantly reduced (*P* < 0.05).

**Fig. 4:**
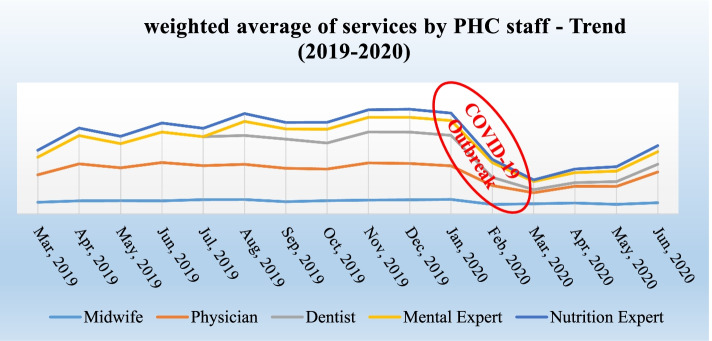
Weighted average of services by PHC staff - Trend (2019–2020)

The number of essential services provided by the physician, dentist, midwife, mental health experts, and nutritionist decreased 2410.65, 2914.66, 460.85, 718.81, 627.95 numbers, respectively (Fig. 4). Based on the results of time trend analysis of the number of essential services provided in PHC facilities since the beginning of 2019 has continued with a relatively stable trend and in some cases fluctuated slightly (*P* > 0.05).

## Discussion

Results of performance analysis of 60 universities of medical sciences across the country in the field of PHC showed that the Covid-19 pandemic has affected the quantities of essential services provided. So that the RSDP is about 1% and the weighted average of the essential services provided by the physician, dentist, midwife, Mental health expert, and nutritionist 2410.65, 2914.66, 460.85, 718.81, 627.95 number, respectively decreased. PCS was the only indicator that did not change before and after the Covid-19 crisis and indicates the upward trend continued.

PHCs are the basic foundation for managing crises and emergencies such as epidemics and health hazards [22, 23]. As evidenced by the Ebola outbreak in West Africa, the strong PHC is essential in preparing for responding to and recovering from emergencies [24]. According to the results of the study, the PHC performance in Iran was affected by the Covid-19 pandemic and the number of provided cares went down significantly during the Covid-19. Studies in other countries, such as China [16] and Sweden [25], also show a declining trend in PHC utilization. According to these studies, there was a significant reduction in the overall utilization of outpatient visits. This issue is a weakness for PHC. It seems that designing and creating a crisis management structure to assess the readiness of the PHC to deal with pandemics and formulate strategies and interventions to prevent decreasing its performance is essential. A strong PHC can be successful by making basic and required care available and providing integrated and coordinated services in early diagnosis and preventing disease outbreaks and preventing unnecessary deaths [26].

Customer satisfaction is one of the most important components in assessing health outcomes and quality in PHC [27]. One of the most important factors that can ensure the quality of PHC during a pandemic is continuous performance monitoring [28]. The results of the current study showed that the level of customer satisfaction has had an acceptable and increasing trend since the beginning of 2019, and the Covid-19 pandemic could not disrupt this trend. According to the results of the studies, the most important factors that affect this indicator include the technical competence of service providers, the cleanliness of the PHC facilities, the physical space, and the approach and communication of health workers [29–31]. In addition to the above factors, kinds of crises and pandemics also can affect the quality of PHC [32].

Mental health is also one of the most important aspects of health. Frequent visits to PHC provider centers due to mental health problems indicate a high prevalence of mental disorders [33]. The stress and anxiety caused by an epidemic, along with the high prevalence of mental health problems in the community, doubled the importance of providing mental health services during a pandemic [34]. Anxiety, overuse of substances, and non-compliance with general hygiene guidelines in people with diseases include the psychological problems caused by Covid-19 disease that have been reported by study [35]. Despite the importance of the issue and the need to increase mental health services, the results of the present study illustrated that the number of mental health services in PHC centers during the Covid-19 pandemic significantly decreased compared to the time before Covid-19. The results of Carolin Hoyer and colleagues also confirm the reduction in mental health service utilization rates during the pandemic [12]. Studies have recommended holding counseling sessions for disease control and management, remote counseling sessions, counseling and psychological support for patients, and prevention of tobacco and substance use, education, and individual psychological interventions during the Covid-19 pandemic [35, 36].

The pieces of evidence show that the distribution of dental problems has changed during the pandemic. After the pandemic, the rate of dental infections has increased and non-urgency cases have decreased [15]. The results of this study also depicted that the rate of dental services decreased considerably after the Covid 19 pandemic. This could be due to the recommendations of the WHO and the other centers for prevention and control of infectious diseases around the world concerning providing non-essential services and as far as possible to provide absenteeism and telemedicine services during the Covid 19 pandemic period [37–39]. Due to its unique features, dental services require requirements and specific clinical guidelines at the time of the infection diseases pandemic according to the Center for Disease Control and Prevention (CDC), dental services should be prioritized during the pandemic and the most important services should be provided. So that minimizes harm to patients due to delays in care and harm to providers and patients due to potential exposure to Covid 19 infection [37].

The covid-19 disrupted all health services utilization except home visits. Nutrition counseling can be done out of facilities through teleconsultation. The role of nutritionists in the prevention and control of Covid-19 disease is essential. Results of many studies have shown that a balanced diet is crucial for building a stronger immune system and reducing the risk of infectious diseases [40, 41]. Notwithstanding, previous studies [42, 43], as well as the results of this study, show that unfortunately after the Covid-19 pandemic the rate of routine nutrition consultations and the provision of appropriate nutritional programs to patients referred to PHC centers, decreased noticeably. The reason for this could be the unknown treatment process of Covid-19 disease at the beginning of the outbreak and the role of nutritional interventions in the treatment of disease, fear of pandemic virus infection, corona restrictions applied by the government to reduce and prohibit traffic and quarantine. Providing online nutrition counseling, telephone calls to patients to control their diet plans, and other non-attendance interventions in times of crisis can improve the performance of the PHC in the field of nutrition services [40, 44].

In the first months of the outbreak; most of the essential services provided in Iran, like other countries, have shifted to services related to Covid-19 disease and PHC providers spent most of their time providing services to Covid-19 patients. Given that There was no information infrastructure to register services related to Covid-19 in the SIB system, so these services were not registered in the system. Although the results of the present study showed that the number of essential services provided in the PHC decreased after the Covid-19 pandemic, Failure to register Covid-19 services in the SIB system could be a major reason for this reduction.

This was a national study that analyzed data from the nationwide PHC. according to the researcher’s best knowledge and literature review, no study in Iran has examined the status of PHC delivery during the Covid-19 pandemic, and this study is the first one to address this issue. However, the present study had two major limitations. The first limitation was calculating the percentage of customer satisfaction based on a general question that may not reflect the various components of the service delivery process. The second limitation was the lack of access to data from other information systems and access to only one data source (SIB System) to examine the purpose of the study. in the Iranian PHC, in addition to the SIB system, there are three other databases, including the PARSA system, health information software entitled “NAB”, and the integrated information system entitled “SINA”, in which the information of 5 universities is registered.

## Conclusion

Covid-19 pandemic affected the performance of PHC in Iran during the pandemic period and reduced the number of essential services provided by physicians, dentists, midwives, nutritionists, and mental health experts. Continuation of the services delivery process after the first 6 months of the pandemic indicates a return to normal trend before the beginning of the pandemic. Failure to register for Covid-19 services, failure to refer to PHC centers at the beginning of the Covid-19 pandemic, application of corona restrictions such as quarantine and curfew at the beginning of the pandemic, stress due to the outbreak of Covid-19 infection, lack of knowledge of effective strategies and interventions for virus management and control along with insufficient use of strong capacities and potentials of PHC to manage and control the Covid-19 crisis can be one of the main reasons for decreasing the number of essential services provided in the early period of the Covid-19 pandemic. Utilizing the experiences and lessons of the Covid-19 pandemic and familiarizing with the capacity of the PHC can help health system managers and policymakers in the possible occurrence of future pandemics and lead to more effective decisions to manage and control the pandemic.

## Supplementary Information

**Additional file 1.**

## Data Availability

All data generated or analyzed during this study are included in this published article.

## Abbreviations

PHC: Primary Health Care
HIS: Integrated Health System
APSD: Actual Percentage of Service Delivery
PCS: Percentage of Customer Satisfaction
