# Supplementary material for: The impact of the Covid-19 pandemic on primary health care utilization: an experience from Iran
Source: BMC Health Serv Res. 2022 Mar 27;22:404. doi: 10.1186/s12913-022-07753-5 (PMC8960210; doi:10.1186/s12913-022-07753-5)
Supplement: Supplementary file 1 — Additional file 1. [file 12913_2022_7753_MOESM1_ESM.docx]

Table 1: Mental health expert Services List

| **Mental Health Expert** | |
| --- | --- |
| **Subjects** | **Weight** |
| In-center group training for the general population and the health team | 16 |
| Off-center group training for the general population | 15 |
| Initial psychological interview | 24 |
| Individual psychological training and interventions | 12 |
| Family psychological education | 12 |
| Complementary screening for tobacco, alcohol and drugs | 13 |
| Complementary screening for spouse abuse | 10 |
| Complementary child abuse screening | 11 |

Table 2: Dentist Services List

| Dentist | |
| --- | --- |
| **Subjects** | **Weight** |
| Double jaw fluoride therapy | 15 |
| Dental fissure sealant | 15 |
| Scaling and brushing of two jaws | 25 |
| Extraction of baby teeth | 15 |
| Permanent tooth extraction | 20 |
| Pulpotomy | 20 |
| Live pulp treatment | 30 |
| Restoration of an amalgam surface | 25 |
| Two-level amalgam restoration | 30 |
| Three-level amalgam restoration | 40 |
| Restoration of a composite surface | 30 |
| Two-level composite restoration | 40 |
| Three-level composite restoration | 45 |

Table 3: Midwifery expert Services List

| Midwifery expert | |
| --- | --- |
| **Subjects** | **Weight** |
| children | 2.03 |
| Teens | 0.7 |
| Youth | 0.69 |
| Middle-aged 30 to 39 years | 1.035 |
| Middle-aged 40 to 59 years | 1.035 |
| Elderly | 1.055 |
| Pregnant mothers | 2.69 |

Table 4: General Physician Services List

| General Physician | |
| --- | --- |
| **Subjects** | **Weight** |
| visit | 10 |
| blood pressure | 3 |
| Diabetes | 3 |
| children | 3 |
| Teens | 1 |
| Youth | 1 |
| Middle-aged | 1.5 |
| Elderly | 1.5 |
| Pregnant mothers | 3 |

Table 5: Nutrition Expert Services List

| Nutrition Expert | |
| --- | --- |
| **Subjects** | **Weight** |
| Nutritional assessment | 3 |
| Providing counseling and training | 2.5 |
| Provide a diet plan | 5 |
